# Using Bayesian Networks to Model Hierarchical Relationships in Epidemiological Studies

**DOI:** 10.4178/epih/e2011006

**Published:** 2011-06-17

**Authors:** Georges Nguefack-Tsague

**Affiliations:** Department of Public Health, Faculty of Medicine and Biomedical Sciences, University of Yaoundé I, Yaoundé, Cameroon.

**Keywords:** Bayesian networks, Hierarchical model, Diarrhea, Disease determinants, Logistic regression

## Abstract

**OBJECTIVES:**

To propose an alternative procedure, based on a Bayesian network (BN), for estimation and prediction, and to discuss its usefulness for taking into account the hierarchical relationships among covariates.

**METHODS:**

The procedure is illustrated by modeling the risk of diarrhea infection for 2,740 children aged 0 to 59 months in Cameroon. We compare the procedure with a standard logistic regression and with a model based on multi-level logistic regression.

**RESULTS:**

The standard logistic regression approach is inadequate, or at least incomplete, in that it does not attempt to account for potentially causal relationships between risk factors. The multi-level logistic regression does model the hierarchical structure, but does so in a piecewise manner; the resulting estimates and interpretations differ from those of the BN approach proposed here. An advantage of the BN approach is that it enables one to determine the probability that a risk factor (and/or the outcome) is in any specific state, given the states of the others. The currently available approaches can only predict the outcome (disease), given the states of the covariates.

**CONCLUSION:**

A major advantage of BNs is that they can deal with more complex interrelationships between variables whereas competing approaches deal at best only with hierarchical ones. We propose that BN be considered as well as a worthwhile method for summarizing the data in epidemiological studies whose aim is understanding the determinants of diseases and quantifying their effects.

## INTRODUCTION

Standard regression methods, including logistic regression and related methods that are commonly used in epidemiological studies do not take account of causal relationships that may exist between the covariates [1-9]. For example, when modeling disease status using a logistic regression, potentially causal relationships between the risk factors are not explicitly modeled. All risk factors are treated as being directly related to disease status; i.e. at the same level of association. The usual procedure is to apply tests of hypotheses, or some model selection criterion, to decide which risk factors should be retained in the model. Causal relationships between some of the risk factors may be already known, or may be regarded as plausible on biological grounds. If so, such information can be, and should be, incorporated in a hierarchical model describing the relationships between disease status and the associated risk factors.

The meaning of "hierarchical" here is not to be taken in the sense of multilevel modeling (or mixed models) where individual patients are grouped, say, by hospital, hospitals are grouped by region, etc.; nor, as in meta-analysis, in which patients are grouped by study. Among other things, by explicitly taking into account of such relationships the ubiquitous problem of multicollinearity can be reduced.

Hierarchical relationships can be represented by arranging variables in a graphical structure called a directed acyclic graph (DAG). An example of a hierarchical model is given by Victora et al. [10]. They consider the presence/absence of an infectious disease in developing countries as a function of several covariates arranged in a hierarchy with 5 levels. The first factor (level 1) is socioeconomic status; level 2 may comprise explanatory variables such as maternal reproductivity and environmental factors; level 3 may have gestational factors; level 4 birth weight and perinatal factors; level 5 child care, diet, nutritional status and previous morbidity factors. The structure is such that the factors at a given level are those that influence the factors at the next level. Finally, some, or all, the above factors may directly affect the risk of a child of acquiring an infectious disease.

By ignoring the hierarchical nature of the relationships in the model, one places risk factors, irrespective of their level, in a single large model and then applies some model selection strategy to eliminate the "non-significant" factors and thereby to select the model that fits the data best in some predefined sense. Victora et al. [10], who point out the inadequacy of such a procedure, proposed fitting a separate model for each level of the hierarchy, namely five individual models.

In order to estimate the effect of a given risk factor using this procedure it is necessary to make adjustments for the confounding role that other risk factors might play in affecting the outcome variable. Other applications of hierarchical models are given in Victora et al. [11], Fonseca et al. [12] for case-control studies, and Nguyen and Nguyen [13] for the determinants of malnutrition.

We propose an alternative unified approach for estimation and prediction, based on Bayesian networks (BNs) that take account of hierarchical structure among covariates. A BN, also known as a Bayesian belief network or belief network, is a probabilistic graphical model tool for describing relationships in a wide variety of domains [14], including various applications in medicine. A medical researcher might develop a BN for diagnosing and for preventing stress fractures. Alternatively a BN could represent the probabilistic relationships between diseases and symptoms. For example, in the diagnosis problem [15,16] the network is used to compute the probabilities of the presence of various diseases, given the symptoms. Nikovski [17] applies BNs to problems in medical diagnosis. Van der Gaag [18] developed methods for eliciting probabilities in a cancer diagnosis study. Lauritzen and Spiegelhalter [19] use BNs to compute the probability of a patient having tuberculosis, lung cancer or bronchitis respectively based on different factors.

We suggest the use of BNs as an alternative to Victora et al.'s approach. It also takes into account the hierarchical relationships among risk factors and disease. An advantage of the proposed approach is that it enables one to estimate the probability that a risk factor and/or the outcome (disease) are in certain states, given the states of the remaining items (risk factors or outcome) in the model. The two approaches are illustrated using a relatively simple model for assessing the impact of three risk factors for diarrhea in a sample of 2,740 children in Cameroon.

## MATERIALS AND METHODS

### Data and variables

Data for 8,096 children aged 0 to 59 months were obtained from the 2004 Cameroon Demographic and Health Survey (DHS) [20]. These are secondary data, made freely available by the National Institute of Statistics (Cameroon) and ICF Macro (Calverton, USA). Ethical issues are covered by the following conditions of ICF Macro: "All DHS data should be treated as confidential, and no effort should be made to identify any household or individual respondent interviewed in the survey. The data sets must not be passed on to other researchers without the written consent of DHS. Users are requested to submit an electronic or hard copy of any reports/publications resulting from using the DHS data files. These reports should be sent to the attention of the DHS data archive, so that it may be forwarded to the country(ies) whose data has(ve) been used".

Our analysis is based on the data for the 2,740 children for whom complete records are available.

The outcome variable of interest here was "Had diarrhea the last two weeks", which is labeled diarrhea (coded 1 if yes and 0 if no). The three covariates considered, labeled sanitation, malnutrition and income, were determined as follows: sanitation indicates the type of toilet facilities that are available (coded 1 if these are good/not shared, and 0 if poor/shared). Although the most common nutritional deficiency affecting the young population in developing countries is insufficient protein and energy intake [21,22], such data were unavailable, and so we used the stunting status (low height-for-age) as a surrogate for malnutrition (coded 1 if the child is stunted, 0 otherwise). The third covariate that, for convenience, we label income, is an indicator of socioeconomic status of households based on wealth index according to DHS methodology. The wealth index takes account household income, use of health services and health status. The observed values of the index were partitioned into quintile groups (coded 1 for the poorest quintile through 5 for the richest).

The statistical analyses were performed with R version 2.10.1 [23] and Hugin Lite version 7.4 [24]. P-values less that 0.05 were considered significant.

### Models

#### The logistic regression approach

All variables were included in a selection procedure. The Akaike information criterion (AIC) [25] selection criterion in a stepwise algorithm was used as variable selection method. Goodness of fit of the models was assessed using the residual deviance.

#### The approach of Victora et al.

Chi-squared tests were used to assess the association between variables. Logistic regressions with diarrhea as response and income, malnutrition and sanitation as predictors were used at each level of the hierarchy (three models).

#### Bayesian networks

A BN is a network of variables or "nodes", each having a probability distribution, connected by directed links, displayed as arrows, that represent causal relationships [26,27]. A variable does not have parent if no links are pointing towards it, and has a parent otherwise. For example, in the structure A→B→C, A has no parent, A is a parent of B, and B is a parent of C. A variable can be either a discrete random variable with a finite number of states, or a continuous random variable (generally assumed to be normally distributed). Associated with a discrete variable is a probability distribution over its states; for a continuous random variables a Gaussian distribution (with given mean and variance parameters) is used instead.

A marginal probability table (MPT) assigns probabilities to the states of variables which have no parents; a conditional probability table (CPT) assigns probabilities to the states of variables which have parents. If a variable with parents is discrete then each entry in its CPT contains a conditional probability for that variable being in a specific state, given a specific configuration of the states of its parents. If a variable is continuous, the CPT contains the (conditional) mean and variance parameters for each configuration of the states of its discrete parents.

If the variable B is the only "cause" of variable A, the CPT for A can be computed using Bayes' rule as


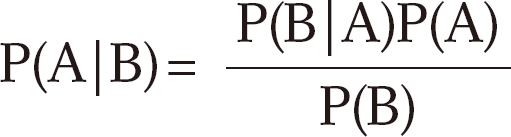


where P(A) is the probability of A and P(B|A) is the conditional probability of B given that A has occurred. If A has K variables, B1, B2,...,BK, as causes (parents), B is replaced in the above formula by B1, B2,...,BK; i.e. a CPT for A is given by P(A| B1, B2,...,BK). The term "evidence" refers to the information available regarding the current state of some of the variables. For example, if one already knows that a child is from a poor family then this constitutes evidence that affects the probability that the child suffers from an infectious disease. It may also affect the probability that the other variables are in given states, for example, that the child suffers from malnutrition. A single item of evidence can affect the entire network. Given evidence E, the CPT for A, given the parent B, is updated using the formula


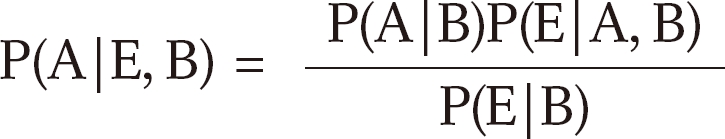


where the left-hand term, P(A|E,B) is called the posterior probability, or the probability of A after considering the effect of the evidence E. The term P(A|B) is called the a-priori probability of A given (only) B. The term P(E|A,B) is called the likelihood and gives the probability of the evidence assuming that A and B have occurred. Finally, the term P(E|B) is independent of A and can be regarded as a normalizing constant, or scaling factor. Details of the use of Bayes rules in BNs can be found in Jensen et al. [28]. Details of the philosophical reasoning and application of BNs can be found in Jensen [26,27].

**Figure 1:**
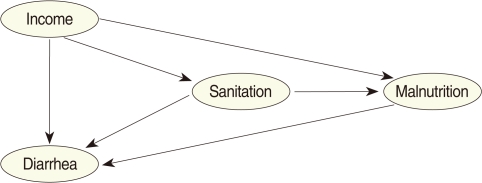
Bayesian network: a simplified conceptual hierarchical framework for diarrhea.

Figure 1 shows a simplified conceptual framework for modeling the diarrhea status of children in our application [10]. It is assumed that income, sanitation and malnutrition are risk factors for diarrhea infection. The model has three levels; income is at the first level, sanitation and malnutrition are at the second level and diarrhea is at the last level. Socioeconomic status (income) affects diarrhea through poor sanitation conditions, and malnutrition, but possibly also through unobserved causes, such as lack of access to health services. That is why we have included an arrow from income to diarrhea. Poor sanitation conditions affect diarrhea directly, due to past infections, and through malnutrition. Malnutrition is a direct cause of disease infections, i.e. a malnourished child is vulnerable to infections such as diarrhea. Cochran-Mantel-Haenszel (CMH) tests were applied to test for conditional independence of variables [29,30]. Conditional independence is a key notion in the construction of a BN. Roughly speaking, two risk factors, which may be highly correlated, are conditionally independent if the association between them is purely the result of their sharing a common cause, say a third factor. The correlation is a consequence of their "common ancestor" (the third variable) and not the result of any direct causal relation between them. Mathematically, two factors are conditionally independent if their conditional distributions, given the level of a third factor, are independent. The notion of conditional independence is used to determine which arrows are essential in the network, and which can be omitted. Thus if there is no arrow between two factors in a BN, then this indicates that the factors are assumed to be conditionally independent.

## RESULTS

A standard logistic regression approach ignores the hierarchical structure of the variables and regards the three covariates as belonging to the same single level. With diarrhea as dependent variable, and assuming that each model includes an intercept, there are seven possible combinations of the three covariates: the probability of having diarrhea depends on 1) income only, 2) sanitation only, 3) malnutrition only, 4) income and sanitation but not malnutrition, 5) income and malnutrition but not sanitation, 6) malnutrition and sanitation but not income, 7) income, sanitation and nutrition.

Using AIC selection criterion in a stepwise algorithm, the logistic regression model LR3 (Table 1) was selected. We also fitted the so-called saturated model, that is the model that includes all possible interactions of the three covariates. The results (not reported here) showed no significant interaction terms.

However, if the hierarchical structure of the data is taken into account, only three models are meaningful [10]. At the first level, income is the only predictor; at the second level, income and sanitation are the predictors, and at the third level, income, sanitation and malnutrition are the predictors. Using the approach of Victora et al., we fitted a logistic regression at each of the three levels (LR1, LR2 and LR 3 in Table 1). Each of these three models fits the data quite well. In each case the coefficient of income is negative and significant, and the coefficients of sanitation and malnutrition are positive and significant.

We now consider the justification for the BN displayed in Figure 1. The associations between degree of freedom (df)=4 and the other variables are all highly significant: sanitation (χ^2^=146.47, df=4, p<0.001), malnutrition (χ^2^=114.26, df=4, p<0.001) and diarrhea (χ^2^=14.53, df=4, p=0.005). Sanitation is strongly associated with malnutrition (χ^2^=10.72, df=1, p<0.001) and with diarrhea (χ^2^=4.96, df=1, p=0.026). Finally, malnutrition is associated with diarrhea (χ^2^=5.60, df=1, p=0.018).

It will be established below that the effect of income on diarrhea is not entirely explained by its indirect effect via sanitation and malnutrition, and so income is likely to be a confounding factor for the relationship between sanitation and malnutrition. Secondly, considering sanitation as an independent risk factor for diarrhea, its association with malnutrition makes it a likely confounding variable for the relationship between malnutrition and diarrhea. These considerations support the BN (Figure 1) based on the conceptual framework designed in Victora et al. [10].

Although the risk factors sanitation and malnutrition are clearly not independent, the null hypothesis that they are conditionally independent, given income cannot be rejected (χ^2^_CMH_=1.07, df=1, p=0.27). In effect sanitation and malnutrition can be regarded as conditionally independent, given income. Thus we could delete the arrow from sanitation to malnutrition in Figure 1, which would simplify the interpretation, but for the purposes of comparison we have not done this. Finally, income and diarrhea are not conditionally independent, given both sanitation and malnutrition (χ^2^_CMH_=15.25, df=4, p=0.004). Thus the arrow from income to diarrhea in Figure 1 is necessary; the effect of income on diarrhea is not entirely explained by its indirect effect via sanitation and malnutrition.

**Figure 2:**
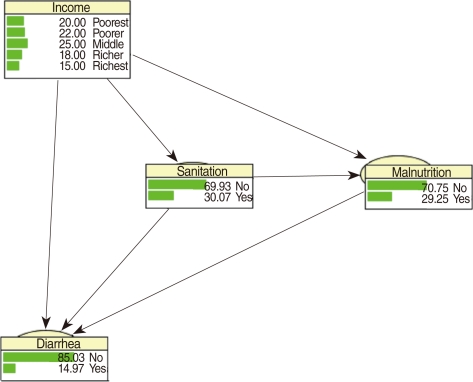
Frequency network showing posterior probabilities (%).

**Figure 3:**
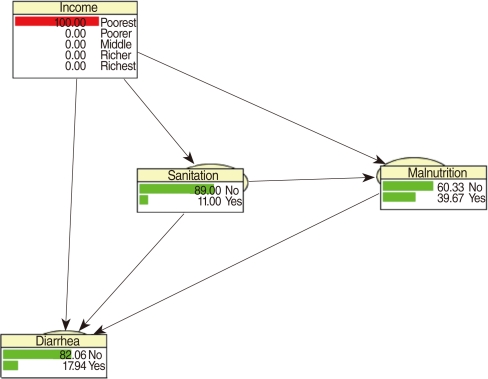
Frequency network showing posterior probabilities (%) when there is evidence that the child belongs to a family in the poorest quintile.

**Figure 4:**
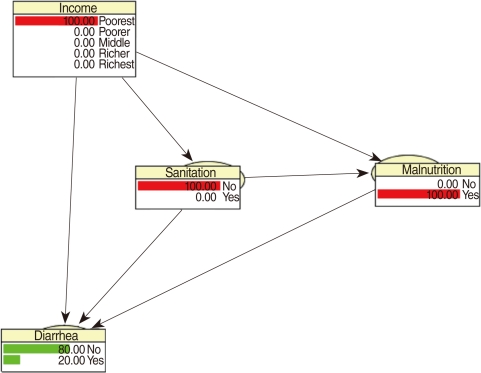
Frequency network showing posterior probabilities (%) of developing diarrhea when there is evidence that the child belongs to family in the poorest quintile, has poor sanitation conditions and is malnourished.

CPTs and MPTs, Tables 2-5 are displayed at each node. Table 6 shows the empirical marginal frequencies of the variables and the adjusted frequencies. The latter take into account the hierarchical structure of the variables; in particular they adjust automatically for any confounding effect. For example, the proportion of children with diarrhea was 15.36%; after taking into account the hierarchical structure, this proportion reduced to 14.97%. In this illustrative application, which is based on a very simple BN having relatively many arrows, the estimates differ very little, but the difference can be substantial in more complex applications in which more risk factors are considered. Figure 2 shows the distributions of the levels of the risk factors and of the disease status taking into account the hierarchical structure. The marginal frequencies estimated using the BN are different but (in this application) very similar to the empirical frequencies. For example the proportion of children living in a poor sanitary condition is 31.24%; but if one takes account of the hierarchical structure and the confounding role of income, this proportion is reduced to 30.07%. The proportion of malnourished children is 30.62%; after taking into account the hierarchical structure and the confounding role of income and malnutrition, this proportion reduces to 29.25%. The distribution of income status does not change because this variable has no parent node. However, it may change when there is "evidence" regarding the state of one or more of the other variables. In general (and as illustrated in Figures 3 and 4) the introduction of evidence regarding the state of any variable can cause all the frequencies of the network to change. Consider, for example, the changes that result from knowing (for certain) that a child's family falls in the poorest group (Note that "evidence" can also be expressed in terms of probabilities, e.g. that there is a 75% chance that the child's family falls in the poorest group). Taking account of this evidence there is a 89.00% probability that the child has poor sanitation, a 39.67% probability that he/she is malnourished, and a 17.94% probability that he/she has diarrhea. Figure 4 shows that if one knows that the child belongs to a family in the poorest group, has poor sanitation and is malnourished, there is 20% probability that he/she has diarrhea.

## DISCUSSION

The variables available in the DHS are obviously imperfect for characterizing income, malnutrition and sanitation [31]. Furthermore, as we have mentioned earlier, causation is in general very difficult to establish [1-9].

For the approach of Victora et al's (the three logistic regression models in Table 1), the coefficient for income, being significantly negative, implies that the probability of being infected with diarrhea decreases as income increases. The coefficient of sanitation, being significantly positive, implies that children with poor or shared toilet facilities are more likely to contract diarrhea than those with good toilet facilities. Likewise, malnourished children were more likely to experience diarrhea.

Model LR1 measures the overall effect of income on diarrhea. LR2 measures the effects of sanitation on diarrhea adjusted for the confounder income; the effect of income is mediated through sanitation. LR3 measures the effects of malnutrition on diarrhea adjusted for the confounders income and sanitation. In the LR3 model the "effect of income" is that which is not mediated via sanitation or malnutrition, and the "effect of sanitation" is that which is not mediated via malnutrition. Thus, a fundamental issue in the above three models is interpretation.

In the BN approach, sanitation and malnutrition, given income, could be regarded as conditionally independent; the relationship between them could be explained purely by the fact that poor families are more likely to have both poor sanitation and malnourished children than are richer families. The fact that income and diarrhea are not conditionally independent, given both sanitation and malnutrition, indicates that socioeconomic status (income) affects the probability of diarrhea in more ways than just via sanitation and malnutrition. It is plausible that it affects that probability via additional unobserved factors, such as lack of access to health services.

It is not the aim of this paper to develop a comprehensive model for the incidence of diarrhea, but rather to illustrate the use of a BN by means of a simple concrete example. The main point is that BNs can provide appropriate and easily interpretable hierarchical structures when the covariates are known to be, or are assumed to be, interdependent in specific ways. The network can be used to predict the state of the variable when there is evidence regarding the state of one or more of the other variables.

BNs can be used in situations where some information is already known and incoming data are partially unavailable [32]. DHSs that are conducted at irregular time intervals are an example of this; BNs can help predicting risk factors/outcomes before complete results for the next survey become available. An additional advantage of BN over Victora et al.'s approach is that it goes beyond hierarchical relationships and can deal with any complex interrelationship between variables, whereas Victora et al.'s approach deals only with hierarchical ones.

Regarding predictions, Victora et al's approach is complicated by the need to first determine which level to choose. For example, in the application considered here, if a child belongs to a family in the poorest group, in order to predict the probability that the child is infected, one must first decide which of the three models (LR1, LR2 or LR3) to use. Secondly that approach only provides estimates of the probabilities for the states of the dependent variable, given the states of the covariates. BNs enable one to estimate not only those probabilities but also the probabilities of the states of those covariates whose states are currently unknown.

The probabilities in the CPTs and MPTs (Table 2-5) can be obtained from survey data (here DHS) or by eliciting estimates from experts (e.g. epidemiologists). Objective survey data and subjective expert assessment can be used either separately or in combination. Of course in the absence of any objective data, elicitation of reliable probabilities is the most difficult aspect in BN modeling. It is especially difficult when many risk factors are being investigated and when these are related in complex ways [33-38]. To alleviate the task, López de Mántaras [33] and van Engelen [34] propose the removal of arcs representing weak dependencies. A key advantage of BNs is the facility of updating (or modifying) the network as new information becomes available. On the other hand, a major criticism of BNs is the need to choose prior probabilities, and (if necessary) to choose appropriate probability distributions.

The BN that we have used for the purpose of illustration is very simple one. There are certainly many factors that affect the probability of diarrhea in addition to those considered here. We also neglected the issue that there were missing data. The little MCAR test [39] (χ^2^=274.83, df=24, p<0.001) suggests that these could be considered as "missing completely at random" (MCAR), i.e no systematic pattern. A more rigorous analysis would necessitate the use of incomplete data methods.

In conclusion, BNs would seem to provide a worthwhile method of summarizing the data in epidemiological studies whose aim is understanding the determinants of diseases and quantifying their effects. The conceptual framework must be clearly set up in order to identify the hierarchical structure in the data. Failure to account for the hierarchical structure of covariates can result in models that lead to unclear, possibly even misleading interpretations of the relationships under investigation, whereas a properly constructed BN automatically corrects for possible confounding variables.

## Figures and Tables

**Table 1:**
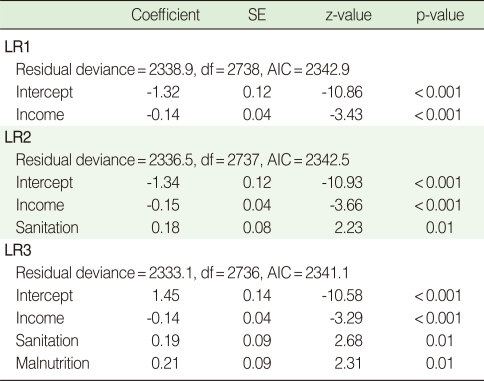
Logistic regression estimates and model summaries

SE, standard error; df, degree of freedom; AIC, Akaike information criterion.

**Table 2:**
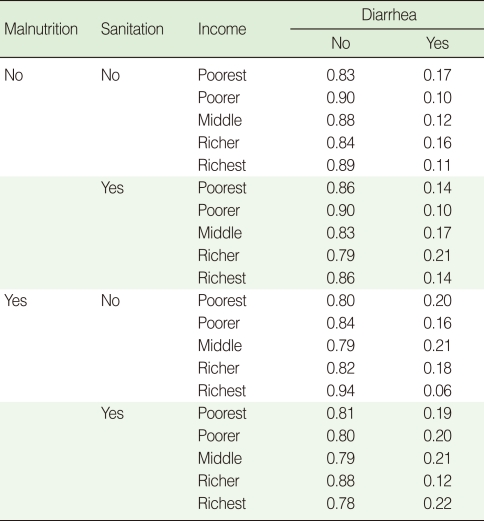
CPT for terminal node diarrhea

CPT, conditional probability table.

**Table 3:**
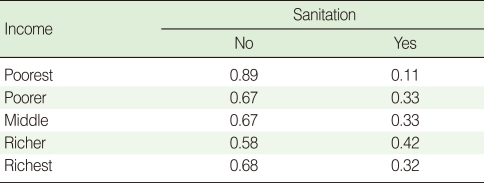
CPT for sanitation

CPT, conditional probability table.

**Table 4:**
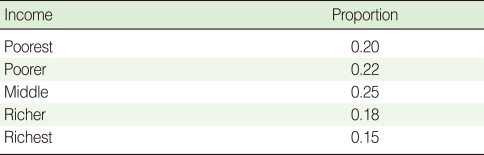
MPT for income

MPT, marginal probability table.

**Table 5:**
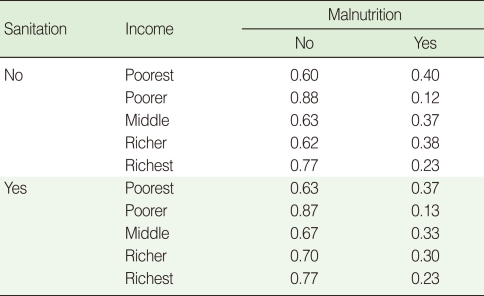
CPT for malnutrition

CPT, conditional probability table.

**Table 6:**
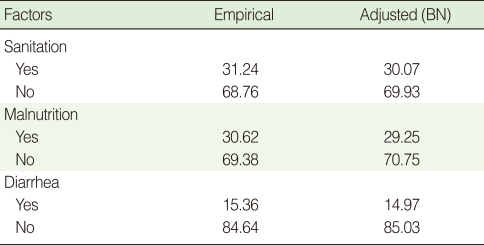
Comparison frequencies (%) from the data and the adjusted frequencies (BN)

BN, Bayesian network.
